# The promising horizon of deep learning and artificial intelligence in flap monitoring

**DOI:** 10.1097/JS9.0000000000000748

**Published:** 2023-09-13

**Authors:** Linjian Liu, Ya Zhang, Xiangjun Xiao, Ruijie Xie

**Affiliations:** a Hengyang Medical School; b Department of Gland Surgery; c Department of Hand and Foot Surgery, The Affiliated Nanhua Hospital, Hengyang Medical School, University of South China, Hengyang, People’s Republic of China

*Dear Editor*,

We recently delved into the enlightening article by Hsu and colleagues in the *International Journal of Surgery* ^1^, which presented a pioneering approach to flap monitoring using a deep learning model integrated application on smartphones. The authors have commendably showcased how this application can adeptly quantify the condition of free flaps, demonstrating its impressive accuracy, discrimination, and calibration capabilities. Their findings underscore the potential of artificial intelligence as a dependable and user-friendly tool for free flap monitoring. The innovative strides taken by Hsu and team in this domain are truly commendable and signify a paradigm shift in how we perceive and utilize technology in medical monitoring.

Free flap monitoring is paramount for the early detection of vascular complications and is proven to enhance salvage rates^2^. As the health conditions associated with aging decline, elderly surgical patients emerge as an especially vulnerable group, making the monitoring of free flaps even more crucial. The quest for the ideal monitoring method is challenging, with criteria ranging from accuracy, reliability, repeatability, and objectivity to cost-effectiveness and user-friendliness^3^. Numerous monitoring tools and techniques have been proposed, each with its inherent strengths and limitations. In this era of technologically advanced medicine, the combination of basic clinical examination with Doppler ultrasound remains the gold standard^4^. However, other techniques, such as the application of wireless transmission and cloud computing technologies, ensure that flap perfusion status can be monitored and assessed anytime, anywhere, offering physicians timely intervention and guidance^5–7^. Coupled with big data training, there’s potential to further enhance the practicality and precision of various traditional flap monitoring methods (Fig. 1). Another noteworthy research trend is the exploration of multicenter collaborations in the medical field^8^. By pooling similar flap cases, a larger volume of data can be amassed, paving the way for more comprehensive training and the development of new models. Furthermore, the accumulation of vast samples could make it feasible to develop flap monitoring models tailored for different demographics, recognizing that patients of varying ages and ethnicities might have significantly different thresholds for vascular obstruction warnings.

**Figure 1:**
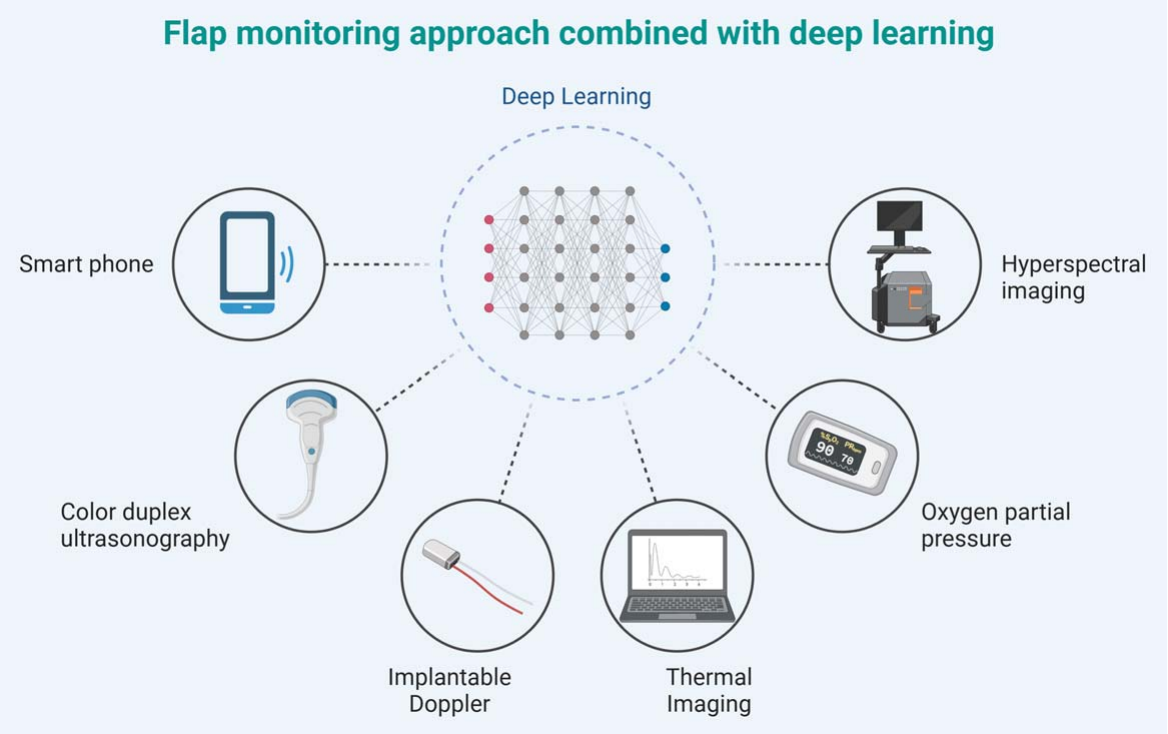
Flap monitoring approach combined with deep learning (created with BioRender.com).

In conclusion, the horizon for deep learning and artificial intelligence in the realm of flap monitoring is vast and promising. The pioneering work of Hsu and colleagues serves as a testament to the boundless possibilities that lie ahead, heralding a future where technology seamlessly intertwines with medical practice to offer unparalleled patient care.

## Ethical approval

Not applicable.

## Consent

Not applicable.

## Sources of funding

The work was supported by the Clinical Research Center of Hand and Foot Wound Repair and Functional Reconstruction in Hunan Province (Grant number 2021SK4030) and the Major Special Project of Hunan Provincial Health and Family Planning Commission (Grant number 20201906).

## Author contribution

All authors read and approved the final version of the manuscript.

## Conflicts of interest disclosure

The authors declare no conflicts of interest.

## Research registration unique identifying number (UIN)

1. Name of the registry: not applicable.
2. Unique identifying number or registration ID: not applicable.
3. Hyperlink to your specific registration (must be publicly accessible and will be checked): not applicable. Acknowledgement None.

## Guarantor

Ruijie Xie

## Data availability statement

The data underlying this article will be shared by the corresponding author on reasonable request. No data were generated during the writing of this study.

## Provenance and peer review

Not applicable.
